# FGF-2-Induced Human Amniotic Mesenchymal Stem Cells Seeded on a Human Acellular Amniotic Membrane Scaffold Accelerated Tendon-to-Bone Healing in a Rabbit Extra-Articular Model

**DOI:** 10.1155/2020/4701476

**Published:** 2020-01-06

**Authors:** Jun Zhang, Ziming Liu, Yuwan Li, Qi You, Jibin Yang, Ying Jin, Gang Zou, Jingfeng Tang, Zhen Ge, Yi Liu

**Affiliations:** ^1^Department of Orthopaedic Surgery, Affiliated Hospital of Zunyi Medical University, China; ^2^Institute of Sports Medicine, Beijing Key Laboratory of Sports Injuries, Peking University Third Hospital, China; ^3^Department of Orthopaedic Surgery, The First Affiliated Hospital of Chongqing Medical University, Chongqing, China

## Abstract

**Background:**

FGF-2 (basic fibroblast growth factor) has a positive effect on the proliferation and differentiation of many kinds of MSCs. Therefore, it represents an ideal molecule to facilitate tendon-to-bone healing. Nonetheless, no studies have investigated the application of FGF-2-induced human amniotic mesenchymal stem cells (hAMSCs) to accelerate tendon-to-bone healing in vivo.

**Objective:**

The purpose of this study was to explore the effect of FGF-2 on chondrogenic differentiation of hAMSCs in vitro and the effect of FGF-2-induced hAMSCs combined with a human acellular amniotic membrane (HAAM) scaffold on tendon-to-bone healing in vivo.

**Methods:**

In vitro, hAMSCs were transfected with a lentivirus carrying the *FGF-2* gene, and the potential for chondrogenic differentiation of hAMSCs induced by the *FGF-2* gene was assessed using immunofluorescence and toluidine blue (TB) staining. HAAM scaffold was prepared, and hematoxylin and eosin (HE) staining and scanning electron microscopy (SEM) were used to observe the microstructure of the HAAM scaffold. hAMSCs transfected with and without *FGF-2* were seeded on the HAAM scaffold at a density of 3 × 10^5^ cells/well. Immunofluorescence staining of vimentin and phalloidin staining were used to confirm cell adherence and growth on the HAAM scaffold. In vivo, the rabbit extra-articular tendon-to-bone healing model was created using the right hind limb of 40 New Zealand White rabbits. Grafts mimicking tendon-to-bone interface (TBI) injury were created and subjected to treatment with the HAAM scaffold loaded with FGF-2-induced hAMSCs, HAAM scaffold loaded with hAMSCs only, HAAM scaffold, and no special treatment. Macroscopic observation, imageological analysis, histological assessment, and biomechanical analysis were conducted to evaluate tendon-to-bone healing after 3 months.

**Results:**

In vitro, cartilage-specific marker staining was positive for the *FGF-2* overexpression group. The HAAM scaffold displayed a netted structure and mass extracellular matrix structure. hAMSCs or hAMSCs transfected with *FGF-2* survived on the HAAM scaffold and grew well. In vivo, the group treated with HAAM scaffold loaded with *FGF-2*-induced hAMSCs had the narrowest bone tunnel after three months as compared with other groups. In addition, macroscopic and histological scores were higher for this group than for the other groups, along with the best mechanical strength.

**Conclusion:**

hAMSCs transfected with *FGF-2* combined with the HAAM scaffold could accelerate tendon-to-bone healing in a rabbit extra-articular model.

## 1. Introduction

Tendon-to-bone interface (TBI)—a key anchor that attaches ligaments/tendons to bones—plays a significant role in relieving the high stress transmitted from the ligament/tendon to the bone [1]. TBI is a special structure between the ligament/tendon and the bone, consisting of four transitional layers: ligament/tendon, uncalcified fibrocartilage, calcified fibrocartilage, and bone tissue [2, 3]. Fibrocartilage is a mechanical load-bearing tissue, and TBI injuries frequently occur in the fibrocartilage area. Upon injury, TBI heals slowly and does not regain its original structure and mechanical property due to the poor healing ability of the fibrocartilage [4]. The regenerative capacity of fibrocartilage is poor and fairly limited owing to its relative avascularity, resulting in very slow recovery of the injured TBI [5]. Recently, many strategies to augment TBI healing have been devised in basic-science researches and clinical treatments, including the use of biomimetic scaffolds, growth factors, and stem cells [6, 7].

The development of tissue engineering technology, involving three pivotal elements—seed cells, growth factors, and scaffold materials—has enhanced the treatment of several diseases [8]. Mesenchymal stem cells (MSCs) are the most commonly used seed cells in tissue engineering technology and include adipose-derived MSCs [9–11], peripheral blood-derived MSCs [12, 13], and bone marrow MSCs (BMSCs) [14, 15]. Owing to their unique advantages, BMSCs have become the most popular seed cells and are now widely used in many fields. However, there are also some disadvantages of BMSCs, such as potential risks of hemorrhage, infection, and immunological rejection response during the harvesting process [16, 17].

Therefore, it is necessary to explore new seed cells that can be applied to tissue engineering technology. Human amniotic mesenchymal stem cells (hAMSCs), harvested from the discarded placenta of healthy puerperant women, have been successfully isolated and used in many studies [18, 19]. Compared with other MSCs, hAMSCs have many advantages, including consistent and wide availability, simple operation, and no ethical controversies [18, 19]. Du et al. reported that hAMSCs had a greater proliferative ability than BMSCs [20], enabling their potential use as seed cells. However, there is limited research about using hAMSCs to accelerate TBI healing [21].

Scaffold materials are the other crucial factors in tissue engineering technology. They not only promote adhesion and proliferation of cells but also have the potential to facilitate cells to differentiate in the desired direction. Human amnion membrane (HAM), the innermost layer of the fetal membrane, is a translucent membrane that has abundant extracellular matrix (ECM), including hyaluronan, fibronectin, and collagens I, III, IV, V, and VII [22–26]. Human acellular amniotic membrane (HAAM) scaffold is a biological scaffold made by decellularizing HAM organization. HAAM scaffold has been widely used as a scaffold to construct engineered tissues and organs owing to its superior characteristics, such as antimicrobial, anti-inflammatory, and nonantigenic properties [22–26].

Many growth factors have been discovered and applied to tissue engineering technology, including transforming growth factor-*β* (TGF-*β*) [27], epidermal growth factor (EGF) [28], vascular endothelial growth factor (VEGF) [29], and fibroblast growth factor (FGF) [30]. Among them, FGF-2 (basic fibroblast growth factor) has been widely used in tissue engineering technology due to its unique advantages [31]. FGF-2 is an effective mitogen for many kinds of cells and has the ability to promote cell proliferation and differentiation [32]. Recently, many studies have found FGF-2 capable of accelerating tendon-to-bone healing [33, 34].

Therefore, in the current study, we hypothesized that (a) FGF-2 could promote chondrogenic differentiation of hAMSCs in vitro and (b) hAMSCs loaded onto the HAAM scaffold and induced by FGF-2 have the potential ability to augment tendon-to-bone healing in vivo.

## 2. Materials and Methods

### 2.1. Isolation, Culture, and Characterization of hAMSCs

For this study, placentas were obtained from the Obstetrics Department of the Affiliated Hospital of Zunyi Medical University with informed consent from each patient before operation. In line with a previous research [35], hAMSCs used in this study were isolated from the human placental amniotic membrane of 5 healthy full-term puerperant women by subjecting to enzymatic (Solarbio, China) digestion twice, followed by collagenase type II (Gibco, USA) digestion once. In brief, the isolated amniotic membrane was minced into pieces and digested twice with 0.05% trypsin/0.01% ethylenediaminetetraacetic acid (EDTA) for 30 mins at 37°C, followed by washing the tissue with phosphate buffer solution (PBS) and digestion with 0.75% collagenase type II for 1 h at 37°C. Third-generation (P3) hAMSCs of good condition obtained by this method were used for subsequent experiments. According to the established protocol [35], the stemness of the hAMSCs isolated from placental amniotic membrane was tested via immunostaining with a stem cell marker and a human amniotic epithelial cell marker, vimentin (1 : 300, ab92547, Abcam) and cytokeratin 19 (CK-19, 1 : 200, ab52625, Abcam), respectively.

### 2.2. Transfection of hAMSCs by Lentivirus Carrying the FGF-2 Gene

Lentivirus containing the *FGF-2* gene was obtained from the Shanghai Jikai Gene Chemical Technology Co. Ltd. According to the manufacturer's instructions, P3 hAMSCs were seeded in a 96-well plate and infected by multiplicity of infection (MOI) of 0, 25, 50, 75,100, and 150. These cells were then classified into three groups: the FGF-2 transfection group, untransfected group, and empty virus group. All three groups were cultured in L-DMEM/F12 culture medium containing 10% (*v*/*v*) fetal bovine serum (FBS, Gibco, USA), 1% (*v*/*v*) penicillin/streptomycin (P/S, Solarbio, China), 1% (*v*/*v*) glutamine (Solarbio, China), and 1% (*v*/*v*) nonessential amino acids (Solarbio, China). The culture medium was changed after 12 h, and hAMSCs were observed under an inverted fluorescence microscope. The optimal MOI value was established by observing the expression of green fluorescent protein (GFP) in the live infected hAMSCs, after which the formal experiment was performed according to the aforementioned method, using the optimal MOI. After 72 h, the expression of GFP was observed under an inverted fluorescence microscope to measure the transfection efficiency. Further, the transfection efficiency was detected using quantitative real-time reverse transcriptase-polymerase chain reaction (qRT-PCR). In brief, total RNA extraction was performed using the RNAiso plus reagent kit (Takara Bio Inc., Shiga, Japan), and cDNA was synthesized using the PrimeScript RT reagent kit (Takara Bio Inc.). The expression of FGF-2 was detected via quantitative real-time PCR using the TB Green Premix Ex Taq kit (Takara Bio Inc.). Human FGF-2 gene was used. The primers used in qRT-PCR are provided in Table 1. Relative gene expression levels were normalized to human glyceraldehyde-3-phosphate dehydrogenase (GAPDH) and calculated using the 2^-*ΔΔ*Ct^ method.

### 2.3. Immunofluorescence Examination and Toluidine Blue (TB) Staining

Immunofluorescence was used to detect the expression of collagen II in each group transfected with and without the *FGF-2* gene. P3 hAMSCs were seeded onto sterile cover slips of 6-well plates at a density of 10^5^ cells/ml (3 wells per group), followed by addition of lentivirus at the optimal MOI. Fourteen days later, after observing intense fluorescence under an inverted fluorescence microscope, the hAMSCs were fixed with 4% paraformaldehyde for 15 min at room temperature. Wells were blocked with 5% goat serum for 60 mins, and the hAMSCs were incubated overnight at 4°C with the primary antibodies against collagen type II (1 : 200, ab34712, Abcam). The next day, hAMSCs were incubated with Alexa Fluor® 594 conjugated anti-rabbit (Abcam, USA) secondary antibodies for 1 hour at room temperature, and cell nuclei were stained using 2-(4-amidinophenyl)-6-indolecarbamidine dihydrochloride (DAPI). The results were observed using an inverted fluorescence microscope. Fourteen days after transfection of the FGF-2 gene, hAMSCs of each group were fixed with 4% paraformaldehyde for 15 mins at room temperature followed by washing thrice with PBS. TB (Sigma, USA) staining was performed, and results were observed under an inverted microscope.

### 2.4. Preparation of HAAM Scaffold

**Figure 1:**
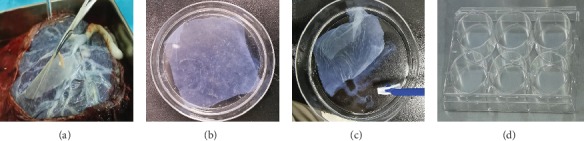
The preparation process of the HAAM scaffold. (a) The HAM was isolated from the placenta. (b) The HAM was decellularized using trypsin. (c) The epithelial cells were removed using a scraper. (d) The HAAM scaffold was put into 6-well culture plates carefully for sterilization.

HAM was isolated under sterile conditions from the placentas to prepare the HAAM scaffold (Figure 1(a)). The isolated HAM was washed with sterile PBS containing P/S solution to separate blood clots, debris, dead cells, and mucus. The HAM was then cut into proper pieces and placed in dishes with the amniotic epithelial layer facing up. Thereafter, the pieces were incubated in 0.25% trypsin with EDTA for 30 mins at 37°C in an incubator (Figure 1(b)). Next, the deciduous epithelial cells were removed carefully with a scraper and were washed thrice with PBS to eliminate the residual epithelial cells and debris thoroughly (Figure 1(c)). The pieces were then recut into smaller sections of approximately 2 × 2 cm size and put into 6-well culture plates carefully (Figure 1(d)). The pieces were then sterilized using ultraviolet radiation and a mixture of P/S and amphotericin for about 30 mins.

### 2.5. Hematoxylin and Eosin (HE) Staining and Scanning Electron Microscopy (SEM) Analysis

HE staining was performed to verify the efficiency of the decellularization process. Fresh HAM and prepared HAAM scaffold were fixed using 4% paraformaldehyde solution for 24 hours, dehydrated, embedded in paraffin wax, and sectioned for HE staining. For SEM analysis, the HAAM scaffold was fixed using 3% glutaraldehyde in 0.1 M PBS for 2 h at 4°C, followed by fixing in 1% osmium tetroxide for 60 mins at room temperature and dehydration using 70%, 80%, 90%, and 100% ethanol for 10 mins. The HAAM scaffold was then air dried, mounted, sputter coated with gold, and tested using the Hitachi SU8100 SEM (Hitachi, Tokyo, Japan).

### 2.6. Cultured hAMSCs and FGF-2-Induced hAMSCs on HAAM Scaffold

hAMSCs and hAMSCs transfected with *FGF-2* were seeded on the completed and sterilized HAAM scaffold at a density of 3 × 10^5^ cells/well. Growth status of the cells was observed daily under an inverted microscope. Additionally, adhesion and growth of the hAMSCs were monitored after the 1^st^ and 7^th^ days via immunofluorescence staining by vimentin. Next, hAMSCs on the HAAM scaffold were fixed with 4% paraformaldehyde and washed with PBS. hAMSCs were blocked using 5% goat serum for one hour and incubated overnight at 4°C with primary antibodies against vimentin, followed by incubation with fluorescent secondary antibodies for 1 h at room temperature. The cell nuclei were stained using DAPI, and the results were observed under a fluorescence microscope. hAMSCs transfected with the *FGF-2* gene were stained with phalloidin to observe cell adhesion and growth after the 1^st^ and 7^th^ days. Subsequently, the cells were washed with PBS and fixed with 4% paraformaldehyde. After permeabilizing the cell membrane with 0.5% polyethylene glycol octylphenol ether (Triton X-100) for 5 mins, TRITC-labeled phalloidin solution (Solarbio, China) was added and incubated for 30 mins. DAPI was used to stain cell nuclei, and the results were observed under a fluorescence microscope.

### 2.7. Surgical Procedure and Creation of Extra-Articular Tendon-to-Bone Healing Model

**Figure 2:**
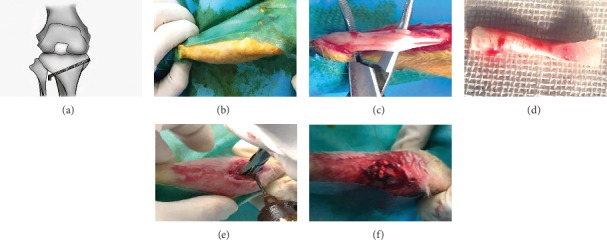
Animal surgical procedure. (a) Brief schematic diagram of the surgery. (b) The skin was sterilized. (c, d) Partial-thickness Achilles tendon of the left hind limb was harvested. (e) Bone tunnel was made in the proximal tibia of the right hind limb. (f) The Achilles tendon graft with four different treatments was passed through the bone tunnel.

A total of 48 healthy New Zealand White rabbits (age 5-6 months; weight 2.5–2.7 kg) were purchased from Chongqing, China. The animal provision license was Animals for Medical Use (Word) No. 2017-0010. The animal study was approved by the Ethics Committee of the Affiliated Hospital of Zunyi Medical College, and all procedures were carried out in accordance with institutional guidelines for the care of animals. Preoperation X-ray examination was performed to exclude relevant diseases that may influence experiment results. Eight rabbits (out of 48) were excluded: two rabbits had fractures during transportation, three had dearticulation, and three had joint deformities. With 5 rabbits in each group, 20 rabbits were used for histological assessment and 20 rabbits were used for radiographic analysis and biomechanical testing. The extra-articular tendon-bone healing model was created according to the established protocol [36, 37] (Figure 2). Briefly, 0.75 ml/kg of 3% sodium pentobarbital was administered intravenously to anesthetize the animals, followed by a middle incision along the Achilles tendon of the left hind limb. A partial-thickness tendon of 2 cm length was harvested as a graft. A bone tunnel of 2.5 mm diameter was made in the proximal tibia of the right hind limb, and the Achilles tendon with four different treatments was passed through the bone tunnel (Table 2).

Next, the ends of the implanted tendons were sutured to the surrounding soft tissue, and 0.5 cm of one end was retained for biomechanical tests to be carried out later. Thereafter, the wounds were sutured layer by layer, and the animals were allowed to move freely without any impediment in their cages after surgery. Penicillin (100,000 U/kg) was injected intramuscularly to prevent wound infection, for three consecutive days postoperation. The rabbits were sacrificed at 3 months after surgery, and graft-tibia complexes (GTCs) were prepared for subsequent tests.

### 2.8. Macroscopic Observation, Morphological Grading, and Imageological Analysis

Three months after the surgery, no infections or other complications were observed in any of the animals. Macroscopic images of the GTC specimens were taken to evaluate the tendon-to-bone healing. Healing status of the tendon-bone interface was assessed using the Yamakado interface morphological grade, which comprised direct type of insertion, collagen fiber continuity, interface without collagen fiber continuity, and separation between the bone and tendon [37]. Each group had five specimens. Additionally, X-ray examination was used to evaluate the bone tunnel area; the GTC samples were scanned perpendicular to the long-bone axis covering the entry and exit of the bone tunnel at the Department of Radiology, Affiliated Hospital of Zunyi Medical University.

### 2.9. Histological Assessment

The GTC samples were fixed in 4% paraformaldehyde solution for 24 h, decalcified using EDTA decalcifying solution (Solarbio, China) for about 2 weeks, and embedded in paraffin for routine histological sectioning. Five-micrometer-thick sections were cut perpendicular to the longitudinal axis of the tibial tunnel and stained with HE, TB, Safranin O/Fast Green, and Masson's trichrome staining.

### 2.10. Biomechanical Analysis

Biomechanical analysis was carried out at the Nanjing BiaoPu Testing Technical Service Co. Ltd. Samples of the GTCs were prepared for biomechanical analysis immediately after sacrifice in accordance with a previously reported protocol [33]. Briefly, all remnant soft tissues, except the transplanted tendon around the bone tunnel, were carefully removed. The transplanted tendon end outside the bone tunnel was sutured for traction, and the tibia was firmly fixed. After a preload with a stationary load of 1 N for 5 min, the biomechanical analysis was performed. The ultimate failure load was carried out with an elongation rate of 2 mm/min. The stiffness and ultimate failure load were measured using the load-deformation curve. For each specimen, the testing was terminated when the graft ruptured or was pulled out of the bone tunnel.

### 2.11. Statistical Analyses

Data were expressed as mean ± standard deviation. Analysis of variance (ANOVA) and Tukey's multiple comparisons were used to determine significant differences. *P* < 0.05 was considered to have statistically significant difference. SPSS software (version18.0; IBM) was used for data analysis.

## 3. Results

### 3.1. Culture and Characterization of hAMSCs

**Figure 3:**
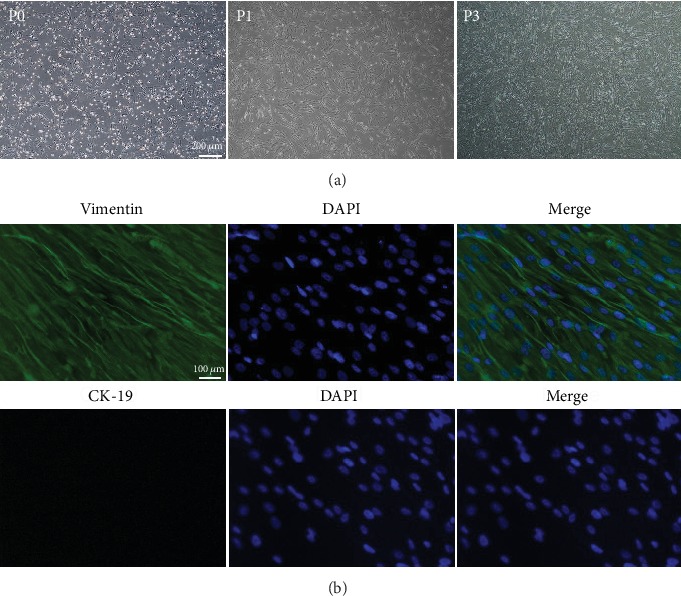
(a) Microscopic observations of P0, P1, and P3 hAMSCs; scale bar = 200 *μ*m. (b) P3 hAMSCs were positive for vimentin. P3 hAMSCs hardly expressed CK-19. Scale bar = 100 *μ*m.

After 48 h in primary culture (P0), the morphology of the hAMSCs exhibited a spindle-shaped exterior under an inverted microscope. After multiple subcultures, the morphology of the hAMSCs gradually became vortex-like and long fusiform (Figure 3(a)). The “stemness” of the hAMSCs was verified via immunostaining to ascertain whether the cells used in this study were indeed stem cells. Immunofluorescence results showed that P3 hAMSCs highly expressed vimentin (MSCs marker) but hardly expressed cytokeratin 19 (a typical phenotype molecule of human amniotic epithelial cells), confirming that the cells used in this experiment were hAMSCs indeed (Figure 3(b)).

### 3.2. Successful Transfection of the FGF-2 Gene into hAMSCs

**Figure 4:**
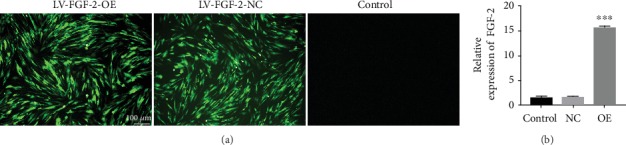
(a) The expression of GFP in each group with lentivirus transfection. MOI = 50. 72 h later, the expression of GFP was observed using a fluorescence microscope. Scale bar = 100 *μ*m. (b) The relative mRNA expression of FGF-2 in each group. ^∗∗∗^*P* < 0.001. Note: OE: overexpression; NC: negative control.

After transfection of *FGF-2* using the optimal MOI value (MOI = 50), cellular morphology and fluorescence expression were observed under an inverted microscope and fluorescence microscope, respectively. The cellular morphology of hAMSCs infected with lentivirus did not change greatly, and insignificant fluorescence expression was observed under fluorescence microscopy after 12 h. With passing time, the expression of fluorescence increased and the strongest fluorescence was observed at 72 h (Figure 4(a)). Next, qRT-PCR was used to assess the transfection efficiency, and its results showed that the mRNA expression of *FGF-2* was statistically higher in the transfected group than in the empty virus group and the untransfected group (*P* = 0.0001). There was no significant difference between the empty virus group and the untransfected group (*P* = 0.06, Figure 4(b)).

### 3.3. Immunofluorescence Examination

**Figure 5:**
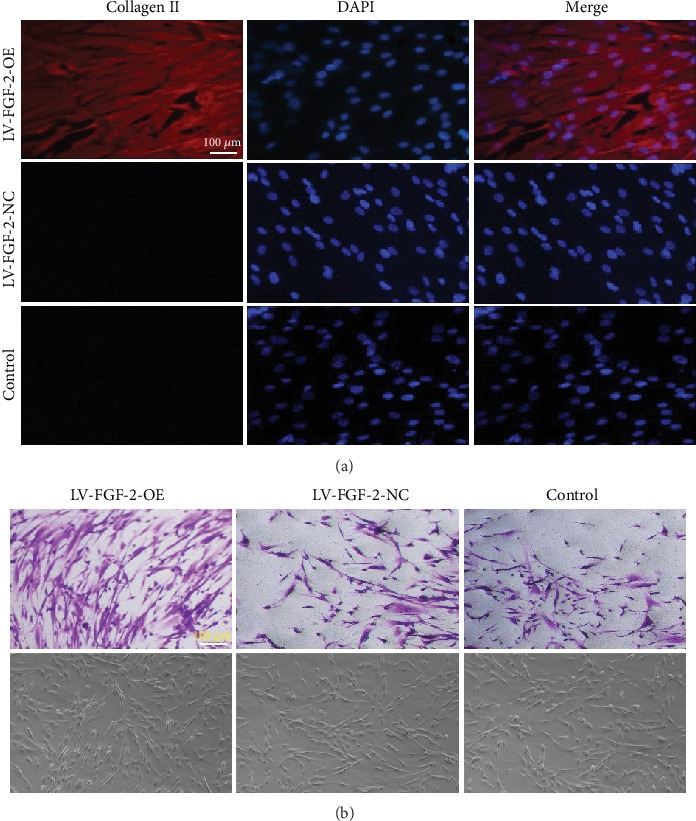
(a) Fluorescence expression of collagen II in each group; scale bar = 100 *μ*m. (b) TB staining of each group; scale bar = 100 *μ*m.

After transfection with lentivirus containing the *FGF-2* gene, immunofluorescence test was used to detect the expression of collagen II in each group, and TB staining was further used to detect chondrogenesis. Immunofluorescence results showed that in the transfected group, collagen II was distributed around the cell nucleus while hardly expressed inside the cell nucleus. However, no fluorescent expression of collagen II was observed in the empty virus group and the untransfected group (Figure 5(a)). These results indicated that FGF-2 could promote and induce hAMSCs into cartilage differentiation. TB staining results showed that there were mass blue-dyed areas; whereas, tiny blue-dyed areas were observed in the empty virus and untransfected groups. These results suggested that FGF-2 had the potential capacity to induce hAMSCs to differentiate into chondrocytes (Figure 5(b)).

### 3.4. HE Staining and SEM

**Figure 6:**
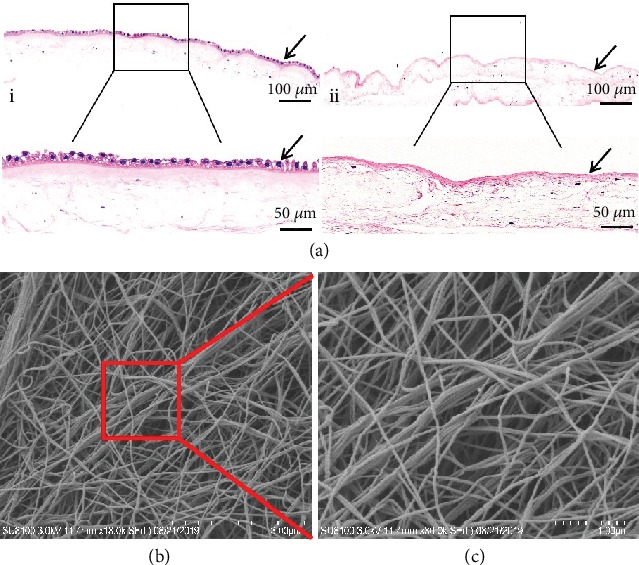
(a) Histological examination of fresh HAM (i) and HAAM scaffold (ii). Magnification of the black rectangular area is displayed at the bottom; scale bars = 100 *μ*m and 50 *μ*m. (b) SEM observations of the HAAM scaffold. Magnification of the red rectangular area is displayed in (c); scale bars = 3 *μ*m and 1 *μ*m.

HE staining revealed that the epidermal layer of the fresh HAM was complete and the nucleus was clearly visible (Figure 6(a), i). However, there were no nuclei in the HAAM scaffold and the epidermal layer disappeared after the trypsin treatment (Figure 6(a), ii). Additionally, the basement membrane of the HAAM scaffold was still intact despite undergoing decellularization treatment, suggesting that the HAAM scaffold maintained the ECM structure favorable for cell adherence and growth. SEM results showed that the HAAM scaffold had a network of spatial structures and there were mass ECM components and collagen components (Figures 6(b) and 6(c)).

### 3.5. Seeding hAMSCs and hAMSCs Transfected with FGF-2 Gene on HAAM Scaffold

**Figure 7:**
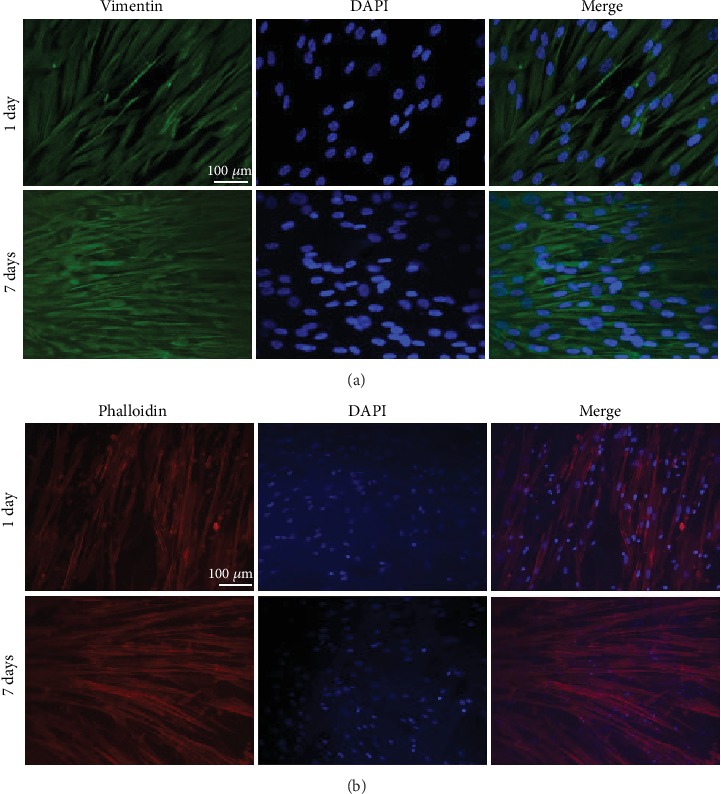
hAMSCs and hAMSCs transfected with the *FGF-2* gene cultured on HAAM scaffold. (a) Immunofluorescence examination using vimentin to observe the adhesion and growth of hAMSCs on the HAAM scaffold; scale bars = 100 *μ*m. (b) Phalloidin staining to test the adhesion and growth of hAMSCs transfected with *FGF-2* gene on HAAM scaffold, scale bars = 100 *μ*m.

One day after the hAMSCs were seeded on the HAAM scaffold, there were only a few hAMSCs on the surface of the HAAM scaffold. After continuously culturing for seven days, the hAMSCs fused into patches covering the surface of the HAAM scaffold. This suggested that the HAAM scaffold had no unfavorable influence on cell proliferation (Figure 7(a)). Similarly, hAMSCs transfected with the *FGF-2* gene showed good adhesion and growth on the HAAM scaffold, evidenced by phalloidin staining (Figure 7(b)).

### 3.6. Macroscopic Observation, Morphological Grading, and Imageological Examination

**Figure 8:**
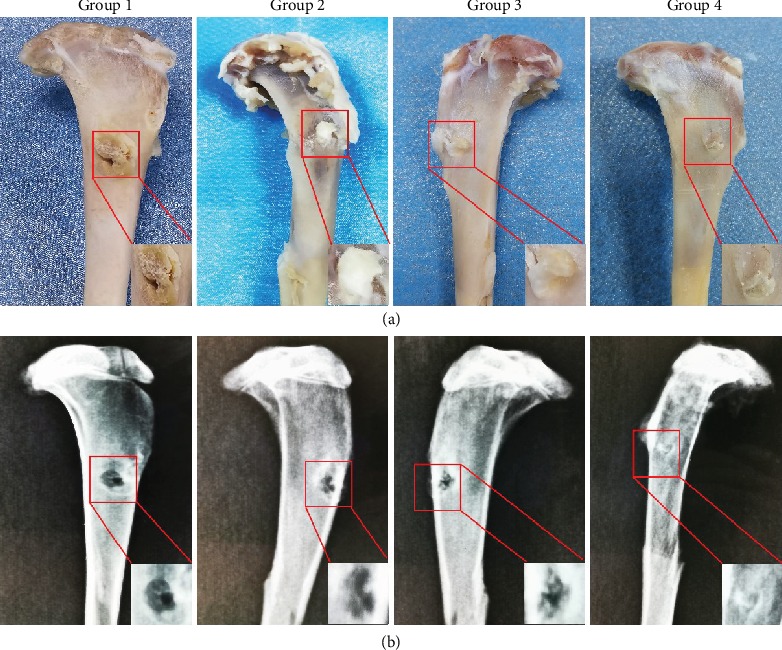
(a) Macroscopic observation of the specimens. (b) Imageological evaluation of bone tunnel in all groups at 3 months postoperation. The magnification of the red rectangular area is displayed in the bottom right corner. Note: Group 1: control; Group 2: HAAM; Group 3: HAAM+hAMSCs; Group 4: HAAM+FGF-2-induced hAMSCs.

Three months postoperation, there were no tears or pullouts from the bone tunnels of the grafts. Poor healing of the tendon-bone interface was observed in Group 1, which was evidenced by obvious gaps between the graft and the bone. In contrast, Group 2 and Group 3 exhibited better healing with marginally narrower gaps. Group 4 displayed the best healing after operation compared with other groups, as there were barely any gaps and the color and luster of the surface were similar to those of normal tissue (Figure 8(a)). Additionally, morphological grading results of the interface showed that direct type of insertion was observed only in Group 4 and Group 4 showed the best healing compared with other groups (Table 3). X-ray examination results showed there was new bone formation observed at the TBI in all the groups. Group 4 demonstrated the highest amount of new bone formation. Additionally, the average bone tunnel area of Group 4 was significantly smaller than that of the other groups. Although, as compared to Group 1, higher new bone formation and smaller average bone tunnel area were observed in Group 2 and Group 3 (Figure 8(b)).

### 3.7. Histological Assessment

**Figure 9:**
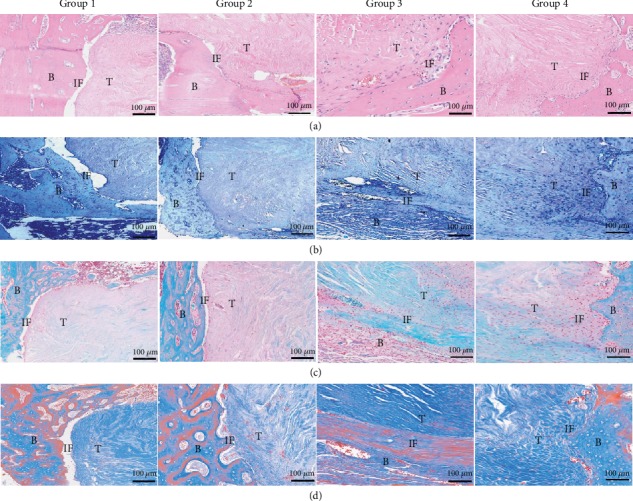
Histological evaluation of TBI in all groups at 3 months postoperation: (a) HE staining; (b) TB staining; (c) Safranin O/Fast Green staining; (d) Masson's trichrome staining. Note: Group 1: control; Group 2: HAAM; Group 3: HAAM+hAMSCs; Group 4: HAAM+FGF-2-induced hAMSCs; B: bone; T: tendon; IF: interface.

HE staining of Group 1 GTCs revealed many inflammatory cells in the tendon-to-bone interface and wide gaps between the grafts and the bone, suggesting that the healing of the tendon-to-bone interface was poor. In contrast, Group 2 and Group 3 exhibited better healing outcomes as compared with Group 1 but lacked any fibrocartilage-like structure. Group 4 showed the best healing, which was evidenced by lack of inflammatory cells, inconspicuous gaps, and mass fibrocartilage-like structures (Figure 9(a)). Further histochemical staining was performed using TB staining. In Group 1, there were some inflammatory cells and evident gaps, consistent with the results of HE staining. Although fewer inflammatory cells and narrower gaps were found in Group 2 and Group 3, no chondrocyte-like cells were observed. In contrast, large areas were stained positively with TB in Group 4, indicating that there was fibrocartilage formation (Figure 9(b)). Safranin O/Fast Green staining was used to further detect fibrocartilage formation in the TBI. These results also showed mass fibrocartilage formation in Group 4 while no fibrocartilage formation in the other groups, evidenced by positive Safranin O/Fast Green staining results (Figure 9(c)). Masson's trichrome staining results revealed vast collagen fibers distributed in the implanted Achilles tendons of Group 4, while only few or no collagen fibers were observed in the other groups, suggesting increased collagen fiber formation in Group 4 (Figure 9(d)).

### 3.8. Biomechanical Analysis

**Figure 10:**
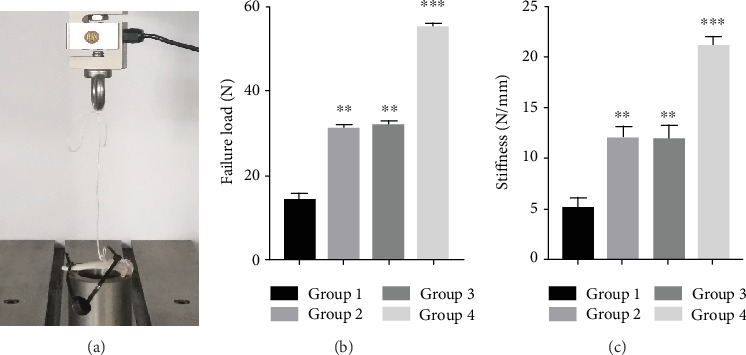
Biomechanical testing. (a) The implementation process of biomechanical testing. (b) Comparison of the ultimate failure load in each group. (c) Comparison of the stiffness in each group. Note: Group 1: control; Group 2: HAAM; Group 3: HAAM+hAMSCs; Group 4: HAAM+FGF-2-induced hAMSCs. ^∗∗^*P* < 0.01 and ^∗∗∗^*P* < 0.001.

Biomechanical analysis was used to investigate the mechanical properties of the grafts three months after surgery (Figure 10(a)). Group 4 showed the highest ultimate failure load. Although the ultimate failure loads of Group 2 and Group 3 were higher than those of Group 1, no statistical difference was found between the two groups (Figure 10(b)). In accordance with the ultimate failure load outcomes, the results of the stiffness test of Group 4 were significantly higher than those of other groups. Similarly, Group 2 and Group 3 showed higher stiffness than Group 1, but there was no statistically significant difference between Group 2 and Group 3 (Figure 10(c)).

## 4. Discussion

TBI is the key attachment site of ligaments/tendons to bones; it not only allows musculoskeletal movements but also has the significant ability to resist high-stress contractions arising from the muscles [38]. However, it is vulnerable to injury due to trauma, overuse, and chronic inflammatory diseases. After injuries, it heals slowly, and the original structure does not restore entirely because of the specialized fibrocartilage zone, which is poor in regenerative ability [39]. Fibrocartilage is the special structure of a normal TBI which plays a crucial role in protecting the TBI from injuries by enabling a gradual transition of mechanical force from the tendon/ligament. The regenerative ability of fibrocartilage is limited owing to its relative avascularity. Although surgical treatments have been used to assist fibrocartilage formation and accelerate tendon-to-bone healing, outcomes have been dissatisfactory. Therefore, the key lies in finding ways to promote fibrocartilage formation by all means available in fundamental researches and clinical settings.

FGF-2 is a potent mitogen for various types of cells and can promote migration, proliferation, and differentiation of MSCs [40]. Owing to its significant role in various types of cell lines, it has been widely applied to restore damaged tissues by promoting cell proliferation, stimulating the release of other growth factors as well as facilitating collagen production. Some studies have found that FGF-2 promotes tendon-to-bone reparation processes, such as healing of ligaments and reconstruction of the rotator cuff, although the exact mechanism is unclear [41, 42]. Additionally, it has been reported that FGF-2 had a positive effect on chondrogenesis and promoting repair of cartilaginous injuries [43].

The HAAM scaffold is a natural biological scaffold, which has been widely used as an extracellular matrix to load cells for the construction of engineered tissues and organs [44]. Compared with other scaffolds, the HAAM scaffold has the following advantages: extensive availability, low cost, low immunogenicity, and no ethical controversies. So far, the HAAM scaffold has been used in many fields, especially in repairing skin defects [45]. In recent years, an increasing number of studies have found that the HAAM scaffold had a positive effect on orthopedic diseases, such as promoting bone regeneration and repairing articular cartilage defects [46, 47].

In this study, we successfully isolated hAMSCs and properties of the MSCs were verified using immunofluorescence staining. The results showed that hAMSCs expressed vimentin—a marker for MSCs—greatly but hardly expressed cytokeratin 19 (a typical phenotype molecule of human amniotic epithelial cells). Although vimentin was used as the only hAMSC marker, other properties of hAMSCs were confirmed in our previous experiment [35], such as plastic adherence, specific surface antigen (Ag) expression, and multipotent ability to differentiate into osteoblasts, adipocytes, and chondrocytes. After confirming MSC properties in our P3 hAMSC culture lines, we used a lentivirus containing the human *FGF-2* gene to transfect the hAMSCs wherein the transfection efficiency was determined via the expression of fluorescence and qRT-PCR. After the *FGF-2* gene was successfully transfected into the hAMSCs, its effect on cartilage differentiation was detected using immunofluorescence staining of collagen II and TB staining. The results demonstrated that FGF-2 could promote the formation of chondrocytes, suggesting that FGF-2 had the potential to induce differentiation of hAMSCs into chondrocytes. Our results are consistent with previous findings that FGF-2 could promote MSCs to differentiate into chondrocytes [48, 49].

We used a simple and inexpensive method to create an HAAM scaffold using enzymatic digestion. In comparison with other methods, this method offered many advantages, such as quicker results, simpler process, and lower cost. Although this method to prepare the HAAM scaffold has been used in other studies [47, 50], we reduced the duration taken for enzymatic digestion, improving the integrity of the extracellular matrix and reducing time and cost. HE staining was performed on fresh HAM and HAAM scaffolds in order to confirm thorough decellularization of HAM. The results showed that epithelial cells existed in the epithelial layer of fresh HAM while no such cells were found in the HAAM scaffold. Additionally, the extracellular matrix remained intact in both the fresh HAM and HAAM scaffolds. These results demonstrated that the decellularization was complete, which was in line with previous studies [44, 50]. The microstructure of the HAAM scaffold was observed using an SEM, revealing that it was porous and suitable for the growth of cells. Next, hAMSCs and hAMSCs transfected with the *FGF-2* gene were seeded on the HAAM scaffold. The adherence and growth of cells on the HAAM scaffold were detected via immunofluorescence staining and phalloidin staining, respectively. The results suggested that hAMSCs and hAMSCs transfected with the *FGF-2* gene could survive and proliferate on the HAAM scaffold, implying that the HAAM scaffold had nontoxic properties.

For in vivo examination, we used a rabbit extra-articular model to explore the effect of the HAAM scaffold combined with hAMSCs transfected with the *FGF-2* gene on tendon-to-bone healing. Three months after surgery, the GTC specimens were harvested and examined. Histological examination showed that Group 4 exhibited better tendon-to-bone healing. We speculate that the following reasons might explain the results. First, FGF-2 transfected into the hAMSCs might have promoted fibrocartilage formation in the TBI in vivo, which could have assisted tendon-to-bone healing. We can surmise this since we had detected that FGF-2 had the ability to promote cartilage differentiation of hAMSCs in vitro. Fibrocartilage is a critical component of TBI [51, 52]; therefore, fibrocartilage formation had a significant role in promoting tendon-to-bone healing. Second, FGF-2 might have acted as a recruiter or stimulant to activate the release of additional significant factors and consequently accelerated tendon-to-bone healing. This speculation was proven by the conclusion that FGF-2 was a vital growth factor in the healing process of injured tissues and was observed in increased quantities after ligament/tendon injuries [52]. Third, the HAAM scaffold might have the potential ability to promote tendon-to-bone healing. Some studies have found that the HAAM scaffold had a positive effect on bone regeneration and bone mineralization [46, 50]. New bone formation is the one of the most important factors of tendon-to-bone healing. Thus, the HAAM scaffold might accelerate tendon-to-bone healing by promoting new bone formation. In practice, the greatest measure of tendon-to-bone healing is the restoration of the original mechanical strength with normal load-bearing capacity. In this study, the highest ultimate failure load and stiffness were detected in Group 4, which demonstrated enhanced mechanical strength and better tendon-to-bone healing.

This study had some limitations. First, the model used in this study was different from clinical tendon-to-bone healing cases, although this animal model had been used in many previous researches [36, 37]. Although this was a not a clinically relevant injury model and had no loading environment that played a significant role in the tendon-to-bone healing, we used this simple model to simulate tendon-to-bone healing and explore the effect of FGF-2-induced hAMSCs combined with the HAAM scaffold on tendon-to-bone healing. Further anterior cruciate ligament reconstruction (ACLR) and rotator cuff (RC) tear models are necessary to further investigate tendon-to-bone healing. Second, only one time interval (three months) was set to evaluate the tendon-to-bone healing in this study, although some researchers also chose only one time point to explore the tendon-to-bone healing [51, 52]. The tendon-to-bone healing is a gradual process, and hence, different time points should be chosen to explore the healing process in further studies. Third, this study was conducted exclusively on an animal model. Considerable efforts will be required to implement this approach in clinical practices. Fourth, we did not use mic-CT to quantitatively evaluate the bone tunnel and the healing status of TBI. In the future, mic-CT and other types of quantitative detection will be used to further assess tendon-to-bone healing.

## Figures and Tables

**Table 1:** Primer sequences for quantitative RT-PCR analysis.

Gene name	Gene symbol	Primer sequence	Accession number
Basic fibroblast growth factor	FGF-2	F: TTCAAGCAGAAGAGAGAGGAG R: TCCGTAACACATTTAGAAGCC	NM_002006.5
Glyceraldehyde-3-phosphate dehydrogenase	GAPDH	F: GCCTTCCGTGTCCCCACTGC R: CAATGCCAGCCCCAGCGTCA	NM_002006.4

**Table 2:** Experimental grouping in vivo.

Groups	Treatment methods
Group 1: control	Achilles tendon with no special treatment
Group 2: HAAM	Achilles tendon wrapped with HAAM
Group 3: HAAM+hAMSCs	Achilles tendon wrapped with HAAM-loaded hAMSCs
Group 4: HAAM+FGF-2-induced hAMSCs	Achilles tendon wrapped with HAAM-loaded FGF-2-induced hAMSCs

**Table 3:** Yamakado interface morphological grade (*n* = 7).

	Group 1	Group 2	Group 3	Group 4
Direct type of insertion	0	0	0	4
Collagen fiber continuity	2	3	3	5
Interface without collagen fiber continuity	3	2	2	0
Separation between the bone and tendon	3	1	1	0

Note: Group 1: control; Group 2: HAAM; Group 3: HAAM+hAMSCs; Group 4: HAAM+FGF-2-induced hAMSCs.

## Data Availability

The data used to support the findings of this study are available from the corresponding author upon request.
